# Emergent adaptive behaviour of GRN-controlled simulated robots in a changing environment

**DOI:** 10.7717/peerj.2812

**Published:** 2016-12-21

**Authors:** Yao Yao, Veronique Storme, Kathleen Marchal, Yves Van de Peer

**Affiliations:** 1 Department of Plant Systems Biology, VIB, Ghent, Belgium; 2 Department of Plant Biotechnology and Bioinformatics, Ghent University, Ghent, Belgium; 3 Bioinformatics Institute Ghent, Ghent, Belgium; 4 Department of Information Technology, iMinds, Ghent University, Ghent, Belgium; 5 Department of Genetics, Genomics Research Institute, University of Pretoria, Pretoria, South Africa

**Keywords:** Complex adaptation, Complex adaptive systems, Self-organizing systems, Artificial life, Swarm robots, Emergent behaviour

## Abstract

We developed a bio-inspired robot controller combining an artificial genome with an agent-based control system. The genome encodes a gene regulatory network (GRN) that is switched on by environmental cues and, following the rules of transcriptional regulation, provides output signals to actuators. Whereas the genome represents the full encoding of the transcriptional network, the agent-based system mimics the active regulatory network and signal transduction system also present in naturally occurring biological systems. Using such a design that separates the static from the conditionally active part of the gene regulatory network contributes to a better general adaptive behaviour. Here, we have explored the potential of our platform with respect to the evolution of adaptive behaviour, such as preying when food becomes scarce, in a complex and changing environment and show through simulations of swarm robots in an A-life environment that evolution of collective behaviour likely can be attributed to bio-inspired evolutionary processes acting at different levels, from the gene and the genome to the individual robot and robot population.

## Introduction

In biology, evolutionary systems of all kinds, such as gene regulatory networks, organisms, populations, and even entire ecological communities can be regarded as complex systems of many interacting components. Furthermore, these interacting components have not evolved independently and in isolation, but in concert throughout different levels of organization. Indeed, gene regulatory networks act in organisms, while organisms form populations and in turn, the latter often form complex ecological communities interacting with other organisms and populations. As a matter of fact, such nested architecture involving different levels of organization can be observed in all biological systems and makes the evolutionary process tick at multiple levels in parallel (Poli, 2001; Mazzocchi, 2010; Wilson, Vugt & O’Gorman, 2008; Koch, 2012). Natural selection and subsequent adaptation is highly dependent on the current environmental and evolutionary context (Allison, 1956; Harper & Pfennig, 2007; Bijma, Muir & Arendonk, 2007; Lichtenstein & Pruitt, 2015). Furthermore, adaptation to a complex environment or to a complex change in environment requires time for interactions between individual entities to evolve gradually. In addition, the more complex a system is, the more unpredictable the outcome of newly introduced changes. Therefore, the complexity of adaptation is often difficult to understand without prior knowledge of the environmental or evolutionary context. Usually, complex adaptation requires more than one novel mutation to yield a functional advantage (Lynch & Abegg, 2010) and even if one single ‘mutation’ could lead to a novel trait (for instance in the case of genetically modified organisms where one gene can be introduced to confer a novel phenotype), the novel trait still needs to exist and persist in a biological context (Williamson, 1992; Prakash, 2001).

Another issue is the ‘cost of complexity’ (Gould, 1988; Orr, 2000; Wagner et al., 2008). Based on Fisher’s geometric model (Fisher, 1930; Orr, 2005), the rate of adaptation decreases quickly with the rise in complexity, because complexity increases the chance of mutations having more pleiotropic effects on the phenotype (Orr, 2000; Lamb, 2000). In addition, it has been suggested that adaptation is easier to be threatened by random changes in organisms or systems with higher complexity (Orr, 2005). All in all, higher complexity requires a finer balance between mutations and adaptive advantage, while it decreases the tolerance for random trial and error (Welch & Waxman, 2003). For the reasons discussed above, it can prove difficult for an evolutionary system to overcome the ‘cost of complexity’ and to achieve novel complex adaptation (Moore, 1955). However, as we observe in the real world, most species can still efficiently deal with these difficulties and develop enough complexity to adapt to ‘their’ environment during evolution (Welch & Waxman, 2003). In our current research, we assume that considering evolution at different levels and considering interactions between these multiple levels of organization (i.e., the genome, the organism, the population, etc.), as found in real biological systems, might be one way to help systems overcome problems associated with the ‘cost of complexity’ (see also Hogeweg, 2012).

To set up a computational framework to study multilevel evolutionary processes and adaptation in complex systems, and to gain further insights into how adaptation in a changing environment might evolve, we have developed a robot controller that combines an artificial genome with an agent-based system that represents the active Gene Regulatory Network(s) or GRN(s). The full regulatory network is encoded in the genome, consisting of both regulatory and structural genes. Depending on the environmental signals or cues, part of the encoded network is activated following the rules of transcriptional regulation. The activated part, modelled by an agent-based system, is responsible for sensing the environmental signals, transducing these signals through the network and translating them into the proper behaviour. Whereas the genome represents the encoding of the transcriptional network, the agents can be seen as the functional gene products (i.e., proteins) of the encoded genes. This way, the agent-based system mimics the active regulatory network and signal transduction system that is also present in naturally occurring biological systems (Yao, Marchal & Van de Peer, 2014).

The use of swarm robots has proved very efficient in many different applications, for example in performing complex tasks in self-assembly (Rubenstein, Cornejo & Nagpal, 2014) self-organizing behaviour (Dorigo et al., 2004), path planning (Ulbrich, Rotter & Rojas, 2016), human–swarm interaction (Nunnally et al., 2016), and cooperative operation (Ducatelle, Caro & Gambardella, 2010; Khaluf, Mathews & Rammig, 2011). In all these studies, swarm robots could achieve complex structures or developed complex behavioural strategies using comparatively simple rules of interaction. Furthermore, in some of these recent studies, swarm robots did not only solve complex tasks, but also unveiled some interesting evolutionary patterns and adaptations. For instance, during simulation, co-evolution has been observed between different robot populations, which eventually would lead to improved adaptation (Nolfi & Floreano, 1998; O’Dowd, Studley & Winfield, 2014; Ferrante et al., 2015).

**Figure 1:**
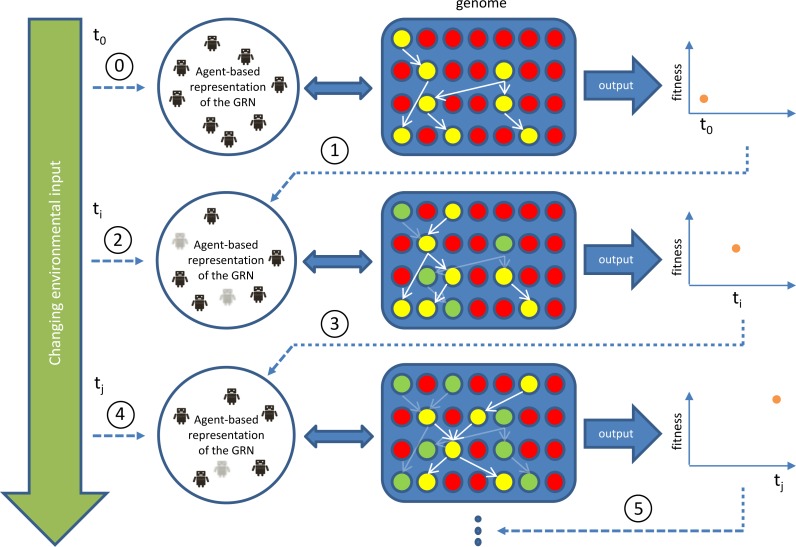
Outline of our GRN based controller during runtime/simulation. The blue area represents the genome with genes indicated as red circles and active (expressed) genes indicated in yellow. Yellow genes connected by white arrows represent the currently active GRN, invoked by environmental inputs. This GRN is translated into (represented by) an agent-based system (Agent-based representation of the GRN). New agents can be introduced/invoked based on changing environmental cues while existing agents will continue to exist or, on the contrary disappear, based on feedback from the system (evaluation of fitness, i.e., energy level), achieved by the currently active GRN. Dark coloured agents (represented by small robot figures) represent active agents, while grey agents represent agents that will be removed at subsequent steps in the simulation. Genes (and GRNs) that are no longer active but reside in the system are denoted by green circles. If a gene is translated into an agent, the ‘concentration’ of this agent, representing dosage, depends on the expression level of the gene, which is determined by the rules encoded in the AG (see Supplemental Information 1). In general, the higher the concentration value of an agent, the higher the influence of the agent on the final output. The concentration of the agent decays with time, mimicking protein degradation. If the concentration of the agent drops below a pre-set minimal level (for instance because of bad performance), the agent will be deleted (e.g., grey agents). The change in concentration of an agent is determined by a default decay rate and its effect on the fitness of the robot.

In our implementation, each individual swarm robot is equipped with a multiple agent controller representing an active GRN, rather than with an Artificial Neural Network (ANN)—based system. In a classic ANN, the architecture of the controller usually allows little interaction with its genetic encoding (the genome). Furthermore, in general, an ANN evolves (is optimized) in response to a specific set of environmental settings and does not easily adapt to changes and/or novel settings. Indeed, ANNs usually need to re-optimize their complete controller network from scratch each time they are subjected to a novel condition (Floreano & Keller, 2010; Le et al., 2013; Mnih et al., 2015). To overcome these shortcomings, also others have previously been inspired to use GRNs as controller systems for robots (e.g., Mattiussi & Floreano, 2007; Joachimczak & Wróbel, 2011; Joachimczak et al., 2012). Using the principles of gene regulation and gene expression, these approaches extend the evolvability of the robot controller, leading to more interesting and complex robot behaviour patterns (Floreano & Keller, 2010; Erdei, Joachimczak & Wróbel, 2013; Joachimczak & Wróbel, 2010). The agent-based GRN controller we developed is similar to the one of Yu & Nagpal (2010). The biggest difference between both systems is that in our system the agents are not only interacting with each other but also with the ‘genes’ (or genome) of each individual robot (Fig. 1). This way, the system is able to continuously produce new agents and GRNs, while in the system of Yu & Nagpal (2010) the agents are defined explicitly by the developer, rather than by ‘evolvable’ genes or genomes. Furthermore, our system uses its genome to store GRNs whose performance was optimized under a particular environmental condition for a sufficiently long time. When subjected to a new environment, the previous condition-specific GRN might become inactivated, but remains present in the genome. This ability to store ‘good behaviour’ and to disconnect it from the novel rewiring that is essential under a new condition allows faster re-adaptation if any of the previously observed environmental conditions is reencountered (Yao, Marchal & Van de Peer, 2014).

In conclusion, we here want to investigate the potential of a novel computational framework that combines a bio-inspired gene regulatory network with an agent-based control system. Through simulations in an A-life environment we show that a swarm of robots running on our bio-inspired controller can indeed evolve complex behaviour and adaptation in a dynamically changing environment.

## Methods

### Rational

The main motivation for the particular design of our framework was to allow artificial evolution at multiple levels thereby mimicking biological evolution more closely. In biological systems, the actual phenotype is not only determined by the GRNs encoded by the genome but also depends on the interacting environment, epigenetic effects, chemical reactions, etc. In general, gene regulation is sensitive to environmental changes and depends on the evolutionary context (Jaenicke & Böhm, 1998; Arnold et al., 2001). Simulating such complex biological evolution with artificial genomes and GRNs is difficult and usually limited by the underlying network structure and the way this is implemented (for instance on an ANN). To encode, represent and maintain a complex evolvable network requires considerable resources which requires (sometimes extreme) simplification of gene regulation and gene regulation modelling. Our agent-based GRN aims to allow adaptation at different levels (so not only at the GRN level) while keeping the system structure as simple as possible. As a result, in our approach, for every gene, we only specified the models about how the particular gene product (agent) interacts with genes, gene products and environmental conditions. Based on a limited knowledge base of interaction rules (which are encoded in the genome), agents are used to maximize the potential of the GRN in a variable evolutionary context.

The experiments are designed such as to test the advantage of integrating evolutionary context at multiple levels (the genome, GRN, organisms, population, …). By comparing with an ANN network based framework for which the evolutionary context at the lower level is limited (the ANN controller has a much simpler implementation of interaction, i.e., there is no interaction between agents such as in the GRN controller, Fig. 1), we can examine the effect of the broader evolutionary context implemented in our current approach, and in particular on emergent behavioural patterns. Through our experimental setup, we hope to demonstrate that adding evolutionary context at the lower level (the genome, the GRN) can accelerate the evolution of complex behaviour at the higher level (population level) while also improving overall adaption in a changing environment (Walker et al., 2004; Ciliberti, Martin & Wagner, 2007; Signor, Arbeitman & Nuzhdin, 2016).

### A GRN based controller

Our GRN-based controller actually consists of two separate layers: a bio-inspired artificial genome (AG), based on the model of Reil (1999), and an agent-based layer. For the genome, in short, we distinguish between signalling, regulatory and structural genes, which all have the same basic structure but different functionalities (see The Artificial Genome, Supplemental Information 1). The AG thus encodes the full regulatory network or entire collection of genes and all its possible interactions, but which specific GRN (part of the AG) is active at a certain time depends on the environmental cues and thus is context-dependent (see Fig. 1). The AG, which consists of 10 chromosomes of 10,000 randomly generated characters, changes over time by evolutionary forces such as mutations and duplications (see Mutational Operators Acting on the Genome, Supplemental Information 1).

The second layer consists of an agent-based system that represents the context-dependent instantiation of the GRN (Fig. 1). Three types of agents have been defined, each corresponding to a specific gene type. Agents can be seen as the translation product of genes. When a new robot is initialised, based on the current sensor inputs, signalling genes will be activated. These genes will produce a number of signalling agents that will produce a signal value. Next, the signalling agents will search the genome to activate those genes for which the signal value matches the binding site. This will lead to the activation of regulatory genes, again via the translation to agents, which are initialized with certain concentration values, mimicking dosage values of proteins or enzymes. Binding of a target gene by a regulator can have different outcomes and will either activate expression of the target gene or block expression of the target gene. If the regulatory gene binds to a structural gene (end product), structural agents will send output values to the actuators, leading to certain behaviour of the (simulated) robot, increasing or decreasing its fitness (Fig. 1). It should be noted that usually, each actuator receives many parameter values from different structural agents and will average these into one final value that will then be used as the control parameter (output value) for this particular actuator. We refer to the Supplemental Information 1 (The Agent-Based System) for more information.

In summary, in our approach, the different components of a GRN, such as regulatory and structural genes, active in a single (simulated) robot, are simulated by different agents. Contrary to fixed nodes in a neural network, these agents can dynamically alter their status and functionality in response to changes in the environment and the feedback they receive based on performance (Fig. 1).

### Simulation framework

We have used artificial life simulation (Sigmund & Young, 1995; Mangel, 1998; Lenski et al., 2003) to see how our GRN-based simulated swarm robots perform in a changing environment. As previously described, every simulated robot has different functionalities, each of which comes with a different energy cost and energy consumption style (see Robot Functionalities, Supplemental Information 1). The total energy consumption for one robot during one time step depends on some basic energy consumption plus some energy consumption for performing certain functionalities. The robots live in a two-dimensional 90 by 90 matrix or grid in which a number of energy (food) sources are randomly distributed. Several types of food sources exist that differ from each other in the minimal amount of energy required to access the food source. If a robot does not have enough energy to cover its basic living energy consumption, it will be regarded as dead and removed from the simulation.

During every time step, every robot will sense the number of surrounding robots and food sources. This information will be returned to the controller of the robot as environmental input (Fig. 1). Except for such external input, the robot will also keep track of its own energy level and energy consumption and of certain other actions such as the number of successful attacks, defences, replications, and so on as internal input. Both external and internal inputs will be regarded as sensor inputs by the controller. Selection and fitness of the robots are all based on energy. The energy consumption of the robot is based on its behaviour. For example, movement, replication, attack and defence all have different costs (see Robot Functionalities, Supplemental Information 1).

In our simulations, we have used both so-called GRN robots (running on the newly developed GRN controller) and ANN robots. For the ANN robots, we also have implemented reinforcement learning on each edge of the ANN to optimize the structure of the ANN dynamically (Lenski et al., 2003; Pfeifer, Lungarella & Iida, 2007; Conforth & Meng, 2008) but we only allow rewiring of the ANN (e.g., setting edge values at zero) based on the feedback of the robot performance. More details of the ANN robots can be found in Supplemental Information 1 (Implementation of the ANN Controller).

During runtime, the robot population will be regarded as gone extinct (or better, not being able to survive) when the population size becomes smaller than 100 (robots). Indeed, we observed that the robot population will quickly die off when the population size is close to 100 and food has become scarce. Although the energy level in the robot population is an indicator of the fitness of the population, there is no explicit fitness function. The energy level of each individual robot is the only critical factor for its survival or reproduction but is not directly responsible for any particular trait to evolve or for showing some behavioural pattern. Indeed, robots can have various strategies to obtain or save energy during the simulation, so the energy level of robots is always a combination of multiple strategies and responses to various environmental conditions.

A similar ecosystem simulation scenario also has been used in previous research, for instance for investigating co-evolution between predators and prey (Nolfi & Floreano, 1998; Erdei, Joachimczak & Wróbel, 2013). However, there are important differences with our approach. For instance, in our simulation, the environment changes continuously as a consequence of the robots’ behaviour. Indeed, the environment dynamically ‘interacts’ with the robots’ behaviour during the entire simulation and adaptations of individual robots can differ depending on the specific context (i.e., number of food sources and number of other robots in close vicinity of the robot). Furthermore, we force the environment to change more drastically by adding fluctuations (every 100 time steps = one season) in the environment mimicking artificial seasons and climate changes in Nature. For instance, during simulation, at certain times (e.g., mimicking harsher winter conditions), all robots have to consume extra energy for movement and survival. The values of these extra energy costs differ at different times during the simulation (e.g., fall and winter are not the same). Every food source is capable to replicate itself (grow) with a certain rate at every time step. The growth rate depends on the surrounding food density (the higher the density, the lower the replication rate). As a result, adaptation of the robots actually comes down to reaching and maintaining a dynamic equilibrium between finding and consuming food and energy balance. Reaching such equilibria is a prerequisite in most, if not all evolutionary scenarios (Rammel, Stagl & Wilfing, 2007). Trying to adapt to a changing environment means that it is difficult to define an explicit fitness function. Every robot needs to adjust its strategy based on the current situation. For example, in our simulations, the energy level of a robot is the key to robot survival and replication but this does not necessarily mean that robots with higher energy levels are the most adaptive. Having more energy to consume more food may undermine new food growth in an environment where food is already scarce. The whole population will face extinction when all food sources are being consumed and therefore, the robots need to evolve more complex behaviour in order to survive for extended periods.

## Results

We have run 100 different simulations, of which 50 with ANN robots and 50 with GRN robots. During our simulations, we specifically analysed the adaptive process regarding the collective behaviour of organisms, based on the interaction between individuals. In our current simulation scenario, where food has to be located, eaten, and thus is becoming scarcer during runtime, we particularly have focused on two kinds of behaviour, namely prey behaviour and a form of cooperation behaviour, where individual robots aggregate to join forces (Sigmund & Young, 1995; Mangel, 1998). Prey behaviour represents the competitive relationship between robots (Nolfi & Floreano, 1998), while the aggregation behaviour represents the symbiotic relationships between individual robots (Yao, Marchal & Van de Peer, 2016). For instance, aggregation, by which energy is shared, can be beneficial for defence against preying. When it comes to preying behaviour, it should be noted that before the predator can actually prey, it needs to perform an attack. An attack action does not always result in preying: if predators are comparatively weaker, an attack action will only cost more energy and will not occur (see Robot Functionalities, Supplemental Information 1).

**Figure 2:**
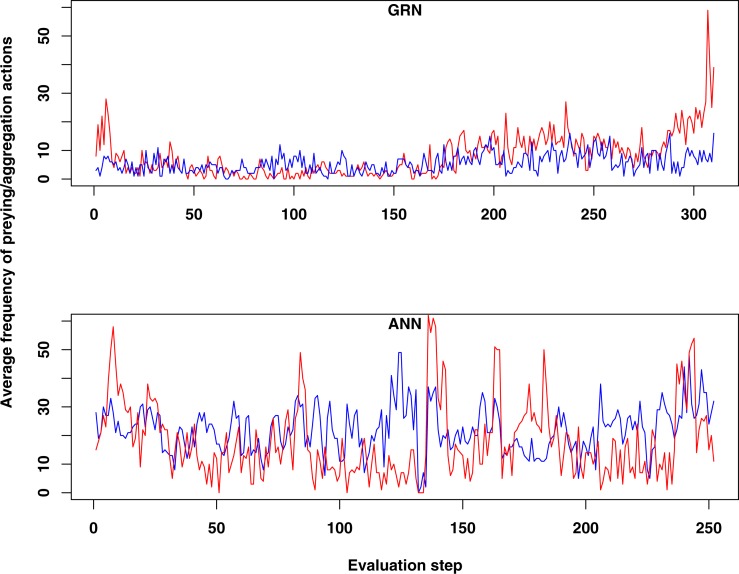
Comparison of the average frequency of successful prey and aggregation actions in GRN and ANN simulations, respectively (evaluated every ten time steps). The average frequency is calculated as the (absolute value/population size)*100. (A) shows the result of GRN robots in the simulation. (B) shows the typical result for ANN robots in the simulation (evaluations have been done every 10 time steps). See text for details.

When we compared the behaviour of GRN and ANN robots, we found some remarkable differences. First, both prey and aggregation/cooperation behaviour occurs more frequently in the ANN robots than in the GRN robots (Fig. 2). However, more or less prey and aggregation behaviour do not necessarily indicate an ‘evolved’ adaptive behaviour. During simulation, GRN robots explore the grid more efficiently than the ANN robots, which reduces the possibility of ‘random’ attacks or aggregation, simply because the robots are more evenly spread over the grid (see further). Maybe a bit unexpectedly, in all simulations (GRN and ANN), cooperation behaviour (in the form of aggregation) does not look very pronounced. We assume that this can be explained by the fact that aggregation requires extra cooperation between multiple robots and such cooperation needs longer to evolve than other kinds of behaviours. This will be investigated in future simulations with longer run times. Furthermore, in our current scenario and implementation, the aggregated robotic organism has all the single robots’ controllers running and the organism is ‘steered’ by the average of all controllers’ output. Moreover, a single robot’s adaptation could be detrimental for the cooperation. Indeed, individual members have evolved their own strategies, but after aggregating, all members of the robotic organism have to use the same strategy. However, a compromise inferred from all individual strategies can be deleterious to most members. To really to be able to evolve interesting aggregation patterns in our evolutionary scenarios and simulations, apart from longer running times, we probably also need to simplify integration and cooperation in the robotic organism. This could for instance be achieved by giving the organism a common controller instead of neutralizing all controller’s outputs.

### Prey behaviour as an adaptation to food scarcity

**Figure 3:**
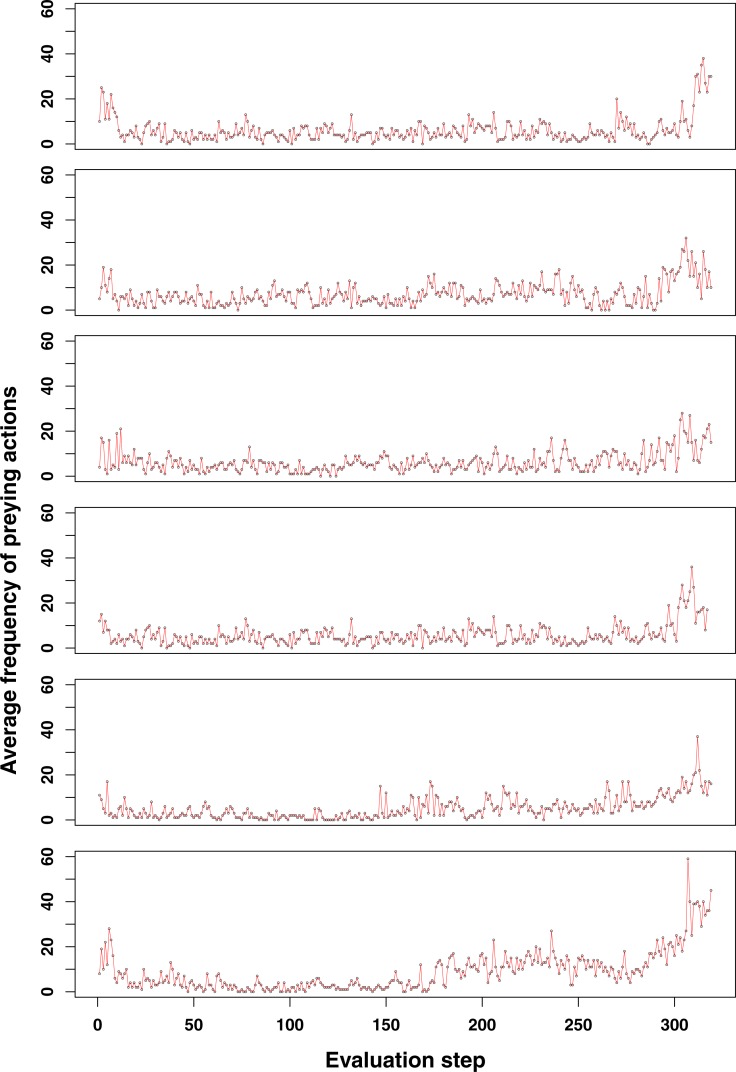
Additional examples of emerging prey behaviour adaptation in the GRN robot simulations. For most of the runs here shown, an increase in the number of preying actions can be observed at the end of the simulation (at time step 3,000). Shortly after however, the population becomes too small and dies (<100 individuals).

For prey behaviour the situation is clearly different. In the GRN robot simulations, but not in the ANN robot simulations, we often observe that the occurrence of prey behaviour increases in the population during the last stages of the simulation, when food becomes scarce (see Figs. 2 and 3). For the ANN robot simulations, prey frequencies are generally higher than in GRN robot populations, but do not seem to be a specific adaptation when food becomes scarcer (not shown). To see whether the increased prey behaviour in GRN robots are indeed a specific adaptation when food becomes scarce, we estimated the Pearson correlation between the prey frequency and the current food number recorded every 10 steps during our simulation. Details on the statistical validation can be found in Supplemental Information 1 (Statistical evidence of preying as specific adaptation).

**Figure 4:**
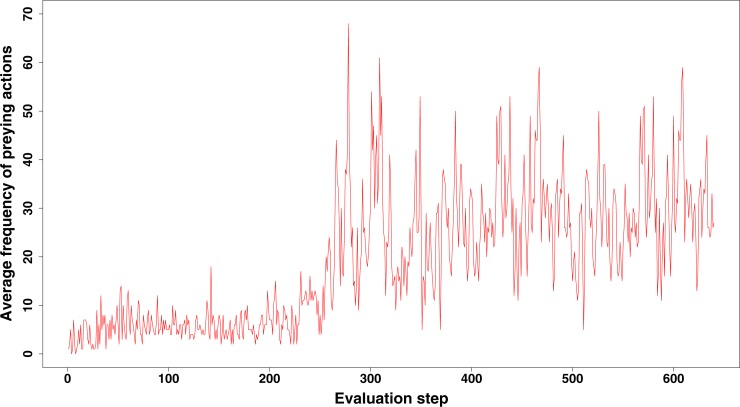
Emerging prey behaviour in the GRN robot simulations. However, unlike as in the experiments shown in Figs. 2 and 3, food is restored (at time step 2,600) before the population dies out. As can be observed, once evolved, prey behaviour continues to be a strategy used by (part of) the population to survive.

The increase of preying behaviour, as seen in a large number of our simulations (see Fig. 2 and more examples in Fig. 3), and which is significantly higher than observed in ANN simulations, likely represents a specific adaptation of the population when food becomes scarce. Prey behaviour reduces the population size but increases the energy of the remaining robots, so at least part of the population can survive longer. On the other hand, prey behaviour also comes with risk and costs energy to every individual robot in the population (defence and attacks both cost extra energy). However, for such complex adaptive behaviour to emerge, a number of conditions need to be fulfilled. Only the combination of a limited number of food sources and large enough population sizes make prey action a trait for selection and therefore an advantageous adaptation. If food sources maintain to be abundant to the population, simply searching for food is obviously an easier and more efficient option than preying. Therefore, we do not observe the combination of prey and flocking together behaviour evolving at the early stages of our simulations when food resources are still abundant. Once preying has evolved, it usually continues to be a useful strategy for the population to survive, as shown in Fig. 4.

### GRN robots have longer life spans

**Figure 5:**
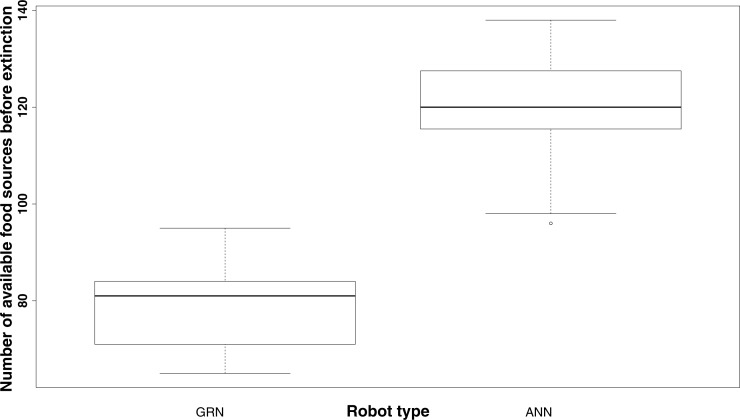
Comparison of the number of food sources left before extinction of the population (sample estimates: mean of GRN = 78.38889, mean of ANN = 119.52174. Significantly different based on two sample *t*-test: *p*-value <2.2e−16). The population is supposed to be extinct when the number of robots drops below 100.

It should be noted that, in general, even without showing prey behaviour, GRN robots survive much longer with fewer food sources than the ANN robots. While on average, the ANN robot population dies when no more than 120 food sources are available, the GRN robot population can survive until the number of food sources has been reduced to, on average, 80 or less (Fig. 5). The fact that the GRN robots can, on average, survive much longer, is, most likely due to the fact that they are more efficient in finding the food sources (exploring the grid), also when these become rarer. Furthermore, GRN robots have higher overall fitness (evaluated based on the energy level) than the ANN robots at similar times in the simulation, supporting their better overall adaptation (Yao, Marchal & Van de Peer, 2014).

**Figure 6:**
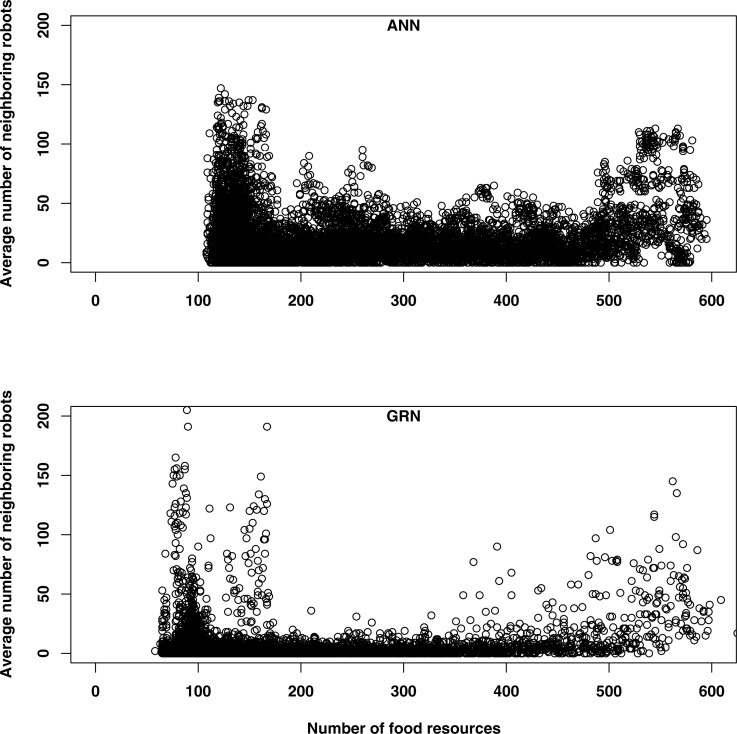
The average number of neighbouring robots (the sum of all neighbouring robots/the number of robots*100) as a function of the number of food sources was used to assess movement of (A) ANN and (B) GRN robots.

When food is abundant, both GRN and ANN robots tend to have more neighbours (Fig. 6). When food is abundant, the robot’s energy level is usually high and this increases the reproduction rates of the robots (reproduction is one of the functionalities of the robots, when their energy levels are high enough). Offspring will be created in the same cells as their parents, which will initially increase the number of neighbours. This explains the higher number of neighbouring robots early in the simulation, for both GRN and ANN robots. When the food becomes scarcer, some robots will stop moving to save energy, while other robots will tend to flock together for improving their prey conditions or for competing for the same food sources. Although both kinds of robots are following basically the same strategies, they will differ in the detailed behaviour. First, the GRN robots spread more efficiently over the grid (see above) and GRN robots have, in general fewer neighbours most of the time (Fig. 6). More efficient dispersion corresponds to wider exploration area and more efficient searching and thus leads to better food searching abilities. Second, when food becomes scarce, GRN robots can start to see having more neighbours (in ANN robots, the number of neighbouring robots throughout the simulation is more constant and the increase in the number of neighbours when food is scarce is much less pronounced). Interestingly, increasing numbers of neighbours correlates with increasing prey behaviour of GRN robots, but only when food is scarce (see above). When food is abundant, such correlation is not observed, which implies adaptation.

**Figure 7:**
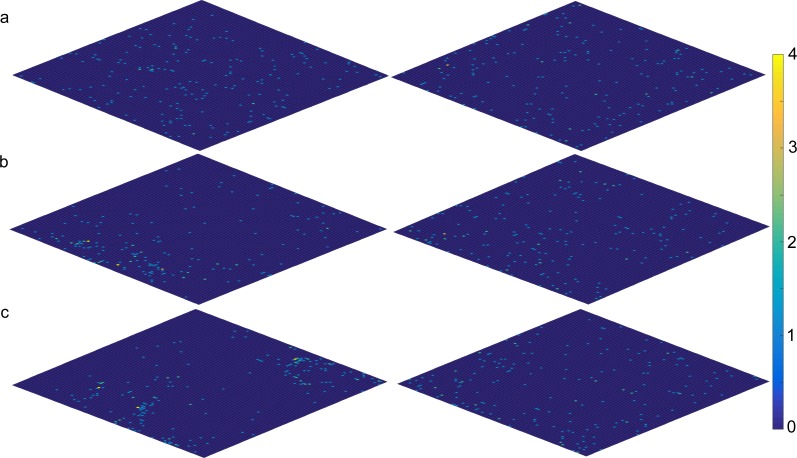
Distribution of GRN (left) and ANN (right) robots during a particular simulation (A) time step 500 (current number of food sources: GRN 339, ANN 461); (B) time step 1,500 (current number of food sources: GRN 208, ANN 267). For the GRN result (left diagram), we can observe ‘colonies’ to be formed; (C) time step 2,500 (current number of food sources: GRN 97, ANN 112). Cells in dark blue represent cells where no robots are present in surrounding cells Cells in light blue have 1 surrounding robot, cells in green have 2, cells in orange have 3, and cells in yellow (max) have 4 surrounding robots (see scale to the right).

Figure 7 shows a comparison, representative for the majority of simulations, of the distribution of GRN robots and ANN robots for one particular simulation and at different time steps. The matrix (grid) corresponds to the simulated environment. Each cell of the map represents one basic space that can be occupied by a robot. All cells are marked in colours dependent on the number of surrounding robots. Cells in dark blue represent cells where no robots are present in surrounding cells, while cells in red represent cells with larg(er) numbers of robots surrounding them. As can be seen, at later stages of the simulation, specific patterns seem to emerge for the GRN robot simulations, while this is much less the case for the ANN robots. However, again it is important to note that, for the GRN robots, the specific robot distribution patterns as shown in Fig. 7, only emerges when food become scarce.

## Discussion

The main aim of our study was not so much to compare the performance of GRN robots and ANN robots (the ANN robots should be seen more as a point of reference), but rather to demonstrate the potential of using GRN robot controllers in studies simulating artificial evolution. However, when comparing our GRN robots with the ANN robots, we observed that the GRN robots survive (much) longer with fewer food sources. There are several reasons for this. First, GRN robots explore the grid more efficiently (Fig. 6; Yao, Marchal & Van de Peer, 2014), while they also seem to evolve alternative strategies, such as prey behaviour (shown in the current study) as specific adaptations to food scarcity. While prey behaviour does occur in the ANN robot population, unlike of what happens in the GRN population it does not seem to be a specific adaptation due to food sources becoming exhausted (see above).

In the simulated environment, there is a basic equilibrium that robots need to reach for survival. In our case, there is a trade-off between availability of food sources and food consumption. Food consumption depends on food searching efficiency, robot population size, and so on. During simulation, new food sources arise (comparable to plant growth, for instance), which is based on the number of the current food sources and is inversely proportional to food consumption and the robot population size. If robots consume the food too fast, food sources will get exhausted rapidly, which will lead to extinction of all robots (the population). On the other hand, if a robot cannot find food fast enough, that particular robot will become weaker and will eventually die of starvation. Therefore, some sort of equilibrium needs to be reached for a healthy population to survive for a considerable amount of time. Furthermore, in our scenario, efficient searching behaviour can help robots to find more food but it also may lead to overly-fast food consumption. In general, searching behaviour will be optimized during the simulation since it directly increases the fitness, as measured by energy level, of individual robots in the short term. On the other hand, to slow down the total food consumption of the whole population, robots can decide to prey other robots to gain energy instead of searching for food, if that would turn out to be the better strategy on the longer term. However, prey behaviour requires an extra energy cost and it requires both the predator and target robot to be in the same cell. Compared with searching for static food sources, finding moving robots and attacking them to gain additional energy will be riskier (i.e., costs more energy) when food sources are abundant. Also, robots can choose to aggregate to share energy and better defend against possible attacks. However, aggregation will also come with a certain cost. Therefore, as already discussed by Smith & Price (1973) organisms need to develop an evolutionarily strategy that is based on their surrounding environment, with equilibrium to change dynamically after environmental changes. As a result, selection in our artificial evolutionary experiments will be more realistic and challenging (Bak & Sneppen, 1993; Spencer & Feldman, 2005; White et al., 2006).

For GRN robots, both the genes (through mutations and duplication) and the GRNs (through the interaction of individual agents) are evolving in the individual robots. As a consequence of their specific structure and implementation, as already stated, GRN controllers will evolve to reach some sort of equilibrium, rather than to evolve or optimize on one single solution. In other words, the GRNs in the robots have a number of dynamic statuses and the transitions between these statuses are based on the interaction of agents, genes and environmental conditions (Fig. 1). The GRN robots directly pass on the environmental information to the particular agents and through feedback and the interaction of these agents at the GRN level, the agents and GRN will remain active as long as performance is good. This way, a self-organized GRN will emerge in the robot. The formation of the GRN itself is a process to reach equilibrium between agents and each individual agent could be seen as part of the task solution. The key goal of the GRN is therefore not to find the optimal solution for a particular environment or task but to reach a dynamic equilibrium in a continuously changing environment. Moreover, when the equilibrium has changed because of a different environmental context, the GRN robots only need to activate and re-organize the corresponding agents instead of evolving all connections again, as would be the case in an ANN. In summary, the agents in our system are dynamically activated or replaced until the GRN reaches a stable status (adaptive status for the robot). When new environmental conditions occur, this will lead to the activation of new agents in the GRN and these new agents may change the current equilibrium. At that time, we will observe a corresponding change in robot behaviour. Later, the agents (both new agents and the already existing agents) will tend to cooperate (exist together) and they will reach equilibrium again. Therefore, behaviour will fluctuate to adapt to changing conditions. Such fluctuations can happen many times until the GRNs in the robots reach a new equilibrium or, alternatively, the robot dies (when not adapted). Therefore, re-balancing processes are inherent to the GRN robot framework. If agents become irrelevant in the current situation, they will be quickly repressed.

For the ANN robots, the whole artificial neural network directly interacts with the environment. The ANN robot’s behaviour can change as well through learning programs. Through evolving the weight parameters of the network that is encoded by the genes, ANN robots may ultimately efficiently reach a good network model for a certain task (Dürr, Mattiussi & Floreano, 2006). However, balancing between multiple tasks (such as preying, defending, replicating) is still hard for an ANN, especially in a continuously changing context and environment, as we have implemented here. In ANN robots, every new environmental change will directly influence selection on the whole network and the selection will only have two possible outcomes: either the rewired network fits the new environment or the rewired network does not fit the new environment.

## Conclusion

Adaptation in the natural environment usually requires cooperation of several traits rather than just one. To allow the evolution of complex adaptation, we adopted a multi-level evolutionary model in the GRN robots. We believe that, using a multiple level evolutionary framework the system can allow different sub-modules (i.e., agents in GRN, or robots in the population) to independently self-organize at a so-called lower level (adaptation), while higher-level evolution is achieved through the interaction between modules across different levels. This will directly encourage the formation of modularity and such self-organized modularity plays a significant role in improving evolvability (Wagner & Altenberg, 1996; Lipson, Pollack & Suh, 2002). Here, we have looked into more detail into the emergence of complex adaptation and show that, using a recently developed GRN robot controller based on an artificial genome encoding agents that represent regulatory or structural genes, complex adaptive behaviour can evolve as a response to a changing environment. Indeed, we observed that, in an evolutionary scenario where food becomes scarce, the simulated robot population often evolves more efficient preying behaviour. Of course, this is something that one could expect to see evolve when food becomes scarce and examples from the real biological world are many—think of the many wars fought for food and other much needed resources throughout history. Interestingly, similar behaviour, which has evolved ‘naturally’ and as an emergent adaptation to a changed environment, can be observed using a swarm of simulated digital life organisms.

##  Supplemental Information

10.7717/peerj.2812/supp-1 Supplemental Information 1 Pseudo code of the GRN-controller and simulation

10.7717/peerj.2812/supp-2 Supplemental Information 2 The relevant raw data and R code for making the figures in this paper

## Additional Information and Declarations

### Data Availability

The following information was supplied regarding data availability: The raw data has been supplied as a Supplemental File.
