# Supplementary material for: Emergent adaptive behaviour of GRN-controlled simulated robots in a changing environment
Source: PeerJ. 2016 Dec 21;4:e2812. doi: 10.7717/peerj.2812 (PMC5180581; doi:10.7717/peerj.2812)
Supplement: Supplemental Information 1 [file peerj-04-2812-s001.docx]

1. **Emergent adaptive behaviour of GRN-controlled simulated robots in a changing environment**

**Yao Yao^1,2,3^, Veronique Storme^1^, Kathleen Marchal^2,3,4,5^, Yves Van de Peer^1,2,3,5^**

^1^ Department of Plant Systems Biology, VIB, Ghent, Belgium

^2^ Department of Plant Biotechnology and Bioinformatics, Ghent University, Ghent, Belgium

^3^ Bioinformatics Institute Ghent, Technologiepark 927, 9052 Ghent, Belgium

^4^ Department of Information Technology, iMinds, Ghent University, Ghent, Belgium

^5^ Department of Genetics, Genomics Research Institute, University of Pretoria, Pretoria, South Africa

Corresponding author: [yves.vandepeer@psb.ugent.be](mailto:yves.vandepeer@psb.ugent.be)

Supplementary Information

**
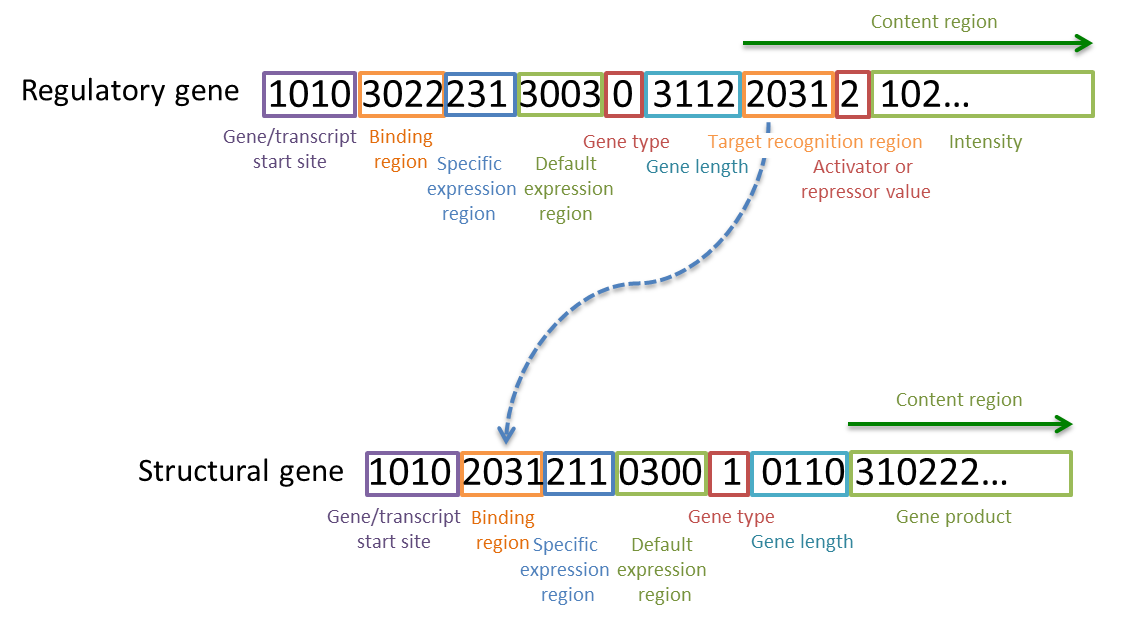
The Artificial Genome (AG)**

**Figure S1. Artificial genome (AG) encoding the core GRN.** Any gene, irrespective of its type, consists of the following components: a transcription start site, a gene identifier (type), a gene length region, a binding site region, an expression level region (default and gene-specific expression region), and a gene content region which is different for structural, regulatory and signalling genes.

The genome structure is based on the model of Reil (1999) and consists of a randomly created string of digits. Genes are not pre-specified, but identified in the randomly built genome as any occurrence of the sequence ’1010’, simulating the concept of a gene/transcript start site, followed by N digits that represent the actual coding gene. The region where no genes are present, i.e. the region between genes, is denoted as intergenic region. Compared to the initial model of Reil, our model was modified as follows (Fig. S1): an explicit distinction is made between signalling, regulatory and structural genes. A gene indicator (one digit) indicates the gene type where 0 denotes that the respective gene has a regulatory function, 1 denotes a structural gene and 2 denotes a signalling gene. Any gene, irrespective of its type, consists of a transcript start site (1010), a sequence specifying gene length (which is intended to avoid overlap between neighbouring genes in the genome) and three other regions that are a binding site region (4 digits), an expression level region (7 digits), which in turn consists of a ‘default’ and a ‘gene-specific’ expression region, and a gene content region. Structural, regulatory and signalling genes further differ in their gene content region. The content region of the regulatory genes consists of three parts: a target recognition site, which defines, in combination with the binding sites, which genes will be recognized and regulated by the regulatory gene, a region defining the regulatory type (being an activator (value 1) or a repressor (value 2) (Fig. S1)) and an intensity region that defines the extent to which the regulator will activate/repress its targets genes (determining gene expression level). The content region of structural genes defines which robot’s actuator (function, see further) the structural gene will influence and also determines the extent to which the gene will influence the actuators (functionality). For signalling genes, the content region encodes a classical ANN structure that receives and integrates signals sensed by the robot. In the current study, the total genome size consists of 10 chromosomes of 10,000 characters.

**Mutational Operators Acting on the Genome**

The AG genome changes over time by evolutionary forces such as mutations (mimicking nucleotide substitutions) and gene duplications.

Mutations (substitutions) are implemented as follows:

In general, the intergenic part of the genome has a higher mutation rate than the ‘coding’ part. The mutation rates are gene specific and are dynamically determined by the fitness of the system: non-functional sequences and genes that have not yet contributed to the individual’s fitness have a default mutation rate Nm (3*10^-5^). However, a gene with a lower contribution to fitness (measured as energy level) will be assigned a higher mutation rate (mimicking a relaxed functional constraint), whereas a gene with a higher contribution has a lower mutation rate (mimicking purifying selection). For each gene, the current mutation rate (Gm) is dependent on both the default non-coding sequence mutation rate (Nm) and an ‘adaptability’ value G1 (contribution to fitness, see also further: ‘The agent based system’), as shown by the following equation:

$$Gm=(1-\frac{G1}{Gmax})*Nm$$

Where Gmax represents the maximum ‘adaptability’ value.

These gene specific evolution models thus mimic the longer term effect of natural evolution in which genes that are under selection pressure tend to be maintained more than genes that are not (purifying selection versus more neutral selection).

Gene duplication is implemented as follows:

There is a software module that, at every time step, will check the expression of all genes and copies the 10 genes with the highest adaptability values (10 genes that contribute most to fitness). When a gene is on the list of genes with highest adaptability for more than 10 time steps, it will be regarded as a gene that qualifies to be duplicated. Like with mutations, the system also has a common background rate for duplications. Every time step, when the system searches for target genes, it also checks whether there is a gap or intergenic region between genes on the genome. If there is a gap (the minimum length being 100 bases), the program will check if there are any qualified genes with a length smaller than the gap and select this one for duplication. When all conditions have been satisfied, the candidate gene will be duplicated (into the gap) and removed from the list of candidates to be duplicated (the gene might get back on the list as long as it keeps its high adaptability value). When the environmental pressure increases, most genes will receive a negative feedback and as a result, the adaptability value of their corresponding agents will decrease. As discussed previously, this will result in an increased mutation rate, which may cause destruction of the promoter region, and consequently the gene downstream of it. Fewer genes on the genome will lead to more gaps and therefore higher duplication rates. More duplicates finally will introduce more variation to the genome, with the possibility of evolving novel GRNs.

**The agent-based system**

Three types of agents have been defined, each corresponding to a specific gene type. Agents can be seen as the translation product of the genes. The agents that correspond to the gene type execute the action defined by the gene type: signalling agents include an embedded ANN, which reads the sensor input values and channel the integrated sensor signals to activate genes in the network. Regulatory agents correspond to regulatory genes, which mediate signal transduction in the network by activating or repressing other regulatory or structural agents according to rules that are defined in the AG. A structural agent will translate the encoded information of a structural gene to an output parameter, which drives the actual actuator (e.g. wheel) of the robot. Each actuator usually receives many parameter values from different structural agents and will average these into one final value that will then be used as the control parameter (output value) for this particular actuator.

If a gene is translated into an agent, the ‘concentration’ of this agent depends on the expression level of the gene (which is determined by the rules encoded in the AG, see above). In general, the higher the concentration of the agent, the higher the influence of the agent on the final output. Once translated, the concentration of the agent decays with time, mimicking protein degradation. If the concentration of the agent drops below a pre-set minimal level, the agent will be deleted. The change in concentration of an agent is determined by a default decay rate and the so-called adaptability value (AV) of the agent.

For each agent, the adaptability value is defined as a combination of the global fitness of the robot (fitness is defined as the overall energy level of each simulated swarm robot) and additional values that express the dependence of the observed fitness on the specificities of a particular set of agents present in the robot at the time its fitness is evaluated. For instance, in our simulations, the adaptability value of a regulatory agent is determined by the global fitness (50%), by the overall average lifetime of the agents (assuming that an ‘agent-set’ with longer average lifetimes will have a greater long-lasting effect on the fitness) (30%), and by the number of agents active in the system, if this number ranges between 30 and 100 (20%). If the number of agents is smaller than 30, we judge the network too small to be viable. If the number of agents is larger than 100, we assume that it is hard to judge on the specificity of each of the agents. Consequently, in both cases we will decrease the contribution of the number of agents to the fitness. The details on how the AV is derived from the global fitness during the simulation can be found here:

The adaptability value of an agent present in a robot at time step i:

V_i_ = ($F_{i}*50\%+A_{i}*20\%+Lf*30\%)*\frac{Ca}{Cmax}$

With Ca = the concentration level (mimicking the amount of protein product) of this agent and Cmax= the maximum concentration level of the same kind agent who ever reached in the previous step.

With Fi being equal to the average normalized change in energy of the robot between step i and i-1: $F_{i}$ = $\frac{E_{i}-E_{i-1}}{\left( E_{i}+E_{i-1} \right)}*100$

E_i_: Energy level of the robot at step $i$; E_i-1_ the energy level of the robot at the previous time step; F_i_: Fitness value of the robot at step $i$

With A_i_ being equal to the number of agents being present in the robot at time step i.

With Lf being equal to the average normalized change in lifetime of the agents present in the robot between time step i and i-1:

Lf = $\frac{L_{i}-L_{i-1}}{{(L}_{i}+L_{i-1})}*100$

L_i_: average lifetime of all agents present in the robot at time step i;

L_i-1_: average lifetime value of all agents present in the robot at the previous time step is

The final adaptability value at time step $i: AV=\frac{V_{i-1}+V_{i}}{2}$

With V_i_ being equal to the adaptability value at time step i and V_i-1_ being equal to the adaptability value at the previous time step.

Assuming the adaptability value of the agent is AV, the feedback effect from that agent on the gene specific expression is at a time step i: Gene specific expression (i) = $10*\frac{AV-20}{100}$ + Gene specific expression (i-1).

**Implementation of the ANN Controller**

Our ANN controller has a fixed fully connected network structure. In the network, there are 350 edges. Each edge corresponds to a gene in the genome. In the ANN controller, between all input nodes, middle nodes and output nodes, each edge corresponds to an agent (that considers reinforcement learning, i.e. ‘learning agent’) that responds to the global fitness of the robot’s Fi in the following way (as determined by its adaptability value).

The adaptability value of the agent present in the ANN at time step i is:

$${AV}_{i}=\frac{F_{i-1}+F_{i}}{2}$$

Two parameters (AVmax and AVchange) will be assigned to each agent. AV_max is a value encoded by the gene and is in the range 0-100. It represents the threshold of being adaptive. If the robot’s performance is higher than this value, the corresponding ANN weight parameter will be always protected from agent rewiring. AV_change is the threshold to decide if the agent will change the corresponding weight parameter. Both parameters could be changed by gene mutations and can differ between different agents. If AVi is smaller than AVmax, the agent will calculate the value AVdistance at time step i:

*AV_distance(i)_ = (AV_max_-AVi)+ AV_distance(i-1)_*

If AVdistance(i) is greater than AVchange, the agent will change the weight parameter at that time step, otherwise the agent will keep the same weight parameter. If AV is greater than AVmax , AVdistance will become 0.

If the agent decides to change the weight parameter (W), it will add or subtract a certain value, based on AVdistance. The value increase C at time step i is determined as:

*C = AV_distance_ *R_change_*

Where Rchange is randomly assigned in the range of 0% to 50%, and the value is encoded in the gene.

The genome of the ANN is a group of parameters which specify the network structure and the corresponding agents on the edges. The mutation rate of the genome is based on the energy level of the robot. The lower the energy level, the higher the mutation rate. The mutation rates ranges from 2*10^-4^ to 0.

The ANN controller is designed in such a way that it matches the GRN controller in its basic functions. The network structure is derived from the artificial genome and therefore we can use the same evolutionary operations to evolve the ANN controller than the GRN controller. To develop an ANN controller that can dynamically adapt to the environment (in a similar way as the GRN controller), we added the ‘learning agent’ on each edge, to allow the ANN to rewire its network structure dynamically based on instant feedback. On the other hand, the ANN controller has a much simpler implementation of interaction (no interaction between agents at the intermediate level, Fig. 1, main paper).

**Robot Functionalities**

In our simulations, each robot has seven different functionalities, each of which comes with a different energy cost and energy consumption style.

The following functionalities are performed by default and not controlled by the output of the structural genes:

1. Sense: every time step, each robot can sense the number of other robots or food sources within a two-cell distance.
2. Prey: the robots can increase their energy level by consuming the food sources located in the same cell as the robot. For different sources of food (see Table S2), the preying might require the robot to possess different amounts of energy in advance. If preying is successful (i.e. the robot has enough energy to take the food), the food will be removed and the energy content of the food will be added to that of the robot.

The following functionalities are driven by the outputs of responsible structural genes (agents):

1. Move: a robot can move to the surrounding cells in the two-dimensional matrix.

Energy cost: 5

Controlling outputs:

Output 1: Value > 0: move forward; Value < 0: move backward; 0 value = no movement

Output 2: Value > 0: go right; Value < 0: go left; 0 value = no movement

1. Attack: Every time step, the robot could choose to attack another robot, which occupies the same cell. If the attack is successful, the attacking robot inherits the energy of the robot that has been attacked and the latter will be removed from the simulation.

Energy cost: 2

Controlling outputs:

Output value 3 >= 0: the robot will try to attack surrounding robots

Output value 3 < 0: the robot will not try to attack surrounding robots

1. Defend: robots can defend against an attack by another robot. This is simulated by investing a certain amount of energy (referred to as defence value). When the robot i is attacked by another robot, its defence value (def_i) plus its energy (Re_i) will be compared with the energy level (Re_h) of the attacking robot. If the attacking robot’s energy is higher (Re_h > def_i+Re_i), the defence will be broken and the defending robot’s energy will be transferred and added to that of the robot that attacks. Otherwise, the defence is successful and costs nothing. Defence values can also be accumulated during the lifespan of a robot.

Energy cost: depends on output 5 (see further)

Controlling outputs:

Output 4 decides on defending behaviour:

Value >= 0: the robot will increase the defence value

Value < 0: no increase of the defence value

Output 5 decides on how much energy is used for defending (only available when output 4 decided on defending):

1. Replicate: when the energy level of robot Re_r_ exceeds a minimal threshold (minR_r_), the robot can choose to replicate. For every time step, the chance of replication (Rc) for a given robot r is based on the following equation:

Rc = $\frac{({Re}_{r}-{minR}_{r})}{500};$

(CurR<MaxR) (CurR: the current number of robots, MaxR: the maximal number of robots).

After replication, the residual energy of the replicating robot will be divided equally over the parent and daughter robots. The new robot will have the same characteristics as the parental robot. When the maximum robot population size has been reached (MaxR), the replication function will be disabled until the population has reduced again.

Energy cost: 1

Controlling outputs:

Output 6: This value determines the threshold for replication (provided the population size has not yet reached a maximal level)

1. Aggregate: At each time step, a robot can send an invitation to another robot. If the invited robot accepts the invitation, both robots will aggregate, merge their energy levels and form a new robot (referred to as a robotic organism). The GRN controllers will be integrated into the new robot by fusing their output signals. At any point, one of the joined robots can stop the aggregation and become single again. Subsequently, the separated robots will receive part of the total energy (total energy divided by the number of aggregated robots in the organism). The advantage of aggregation is that the joined ‘robotic organism’ will have more energy and greater defence capabilities. For example, the maximum energy of a robotic organism (including n robots) will be equal to $\sum_{n=1}^{n} Maxe\left( n \right)$ (Table S1). Such robotic organism will perform better on preying, defence and attacking. The disadvantage of aggregation is that, for every time step, it comes with an energy cost (see Table S1).

Energy cost: 1

Controlling outputs:

Output 7 decides whether to aggregate with another robot

Output 7 Value >= 0: the robot will aggregate with the surrounding robot value

Output 7 Value < 0: the robot will not aggregate with the surrounding robot

Output 8 Value: determines whether to disassemble (only available when the robot is part of a robotic organism):

Output 8 Value >= 0: the robot will stay aggregated

Output 8 Value < 0: the robot will disaggregate

The total energy consumption for one robot during one time step is described by the following equation (with n corresponding to the number of functionalities in time step i):

For a single robot:

Total energy consumption =$\sum_{i=1}^{n} {Ae}_{i}+Be$

For a robot that is part of a robotic organism (aggregate of robots):

Total energy consumption = $\sum_{i=1}^{n} {Ae}_{i}+Be+Ee$,

With *Ae* being the energy consumption for actions, Be being the basic energy consumption required for each time step, and Ee being the extra energy consumption for aggregation during each time step.

**Statistical evidence of preying as specific adaptation**

To see whether the increased prey behaviour in GRN robots are indeed a specific adaptation when food becomes scarce, the Pearson correlation was estimated between the prey frequency and the current food number recorded every 10 steps during our simulation. This was done for all GRN and ANN simulations with the cor.test function from the stats package in R (R Core Team, 2015).

In addition, the prey frequencies recorded until the current food number had dropped to 300 (referred to as the ‘plenty food’) were compared with the prey frequencies recorded until the current food number was dropped to 0 (referred to as the ‘low food’ group’). The average prey frequencies in both groups were compared with a one-sided two-sample t-test with unequal variances (as tested with an F-test). This was done for all GRN and ANN simulations with the var.test and t.test function in R. The two groups were considered to have a significant different mean at a family-wise error rate of alpha=0.05 when the p-value < 0.001 using Bonferroni’s correction for multiple testing. At last, to verify whether the average prey frequency was higher for the GRN robot simulations than for the ANN robot simulations, autoregressive moving average (ARIMA) models were fit to each simulation from the point where the food number of 300 was reached onwards. For all simulations, arima models (4,1,4) were fit to the data including a drift term with the Arima function from the R package forecast (Hyndman and Khandakar, 2008; Hyndman, 2015) to test whether the mean of the series of prey action in the GRN simulations did indeed shift more upwards with time than in the ANN simulations. To this end, the 50 estimated drift values from the GRN simulations were compared with the 50 estimated drift values from the ANN simulations with a one-sided two-sample t-test with equal variances.

The average Pearson correlation between the prey action and the current food number was

-0.019 for the ANN simulations and -0.234 for the GRN simulations. There was thus a slightly negative correlation in the GRN simulations, as one would expect, while there was none in the ANN simulations. This is also shown with the comparison of the two food groups (‘plenty-food’ versus ‘low-food’). In 44% of the GRN simulations, the average prey action in the ‘low-food’ group was significantly higher than in the ‘plenty-food’ group. This was only true for 28% of the ANN simulations. There is thus a tendency of an increase in prey action over time in the GRN simulations (average drift is 0.03). This is significantly higher than in the ANN simulations where the average drift is negative (-0.06, p < 0.05).

# Appendix A: Pseudo code for the main simulation loop:

Int I = the number of alive robots;

Int N= the number of all cells on the map;

Int T=current time step;

Int A=0;

int main()

{

Initialize the robots and food sources on the map;

While (I>=100||T<=500)

{

Update T;

If (T%100==0)

{

Set environmental parameters for season A;

A++;

}

If (A>3)

Set A=0;

For (i = 0; i <N; i++)

{

Check remaining food and decide whether food sources can grow;

}

For (i = 0; i <N; i++)

{

Check all robots and Run all robot’s sub-loops;

Update foods and environment;

}

For (i = 0; i <I; i++)

{

Referee the interaction behavior of all robots;

Evaluate new status of each robot and store the information;

}

If (T%10==0)

{

For (i = 0; i <I; i++)

{

Output robot information to files;

}

}

End while;

}

}

Pseudo code for the single robot in simulation:

Int E=the current energy level of robot;

Int L=the life time of the robot (100);

bool Create;

Run LoadGenome(Create)

{

If (Create==true)

Function that randomly creates AG (when initializing the simulation);

else

{

Copies the artificial genome from other robot (when robots produce offspring);

Running a mutation operation on the genome;

}

}

Run robot loop at every time step

If (E>0)

{

L--;

If(L<0)

Call Delete function and out of the loop;

Update the fitness of the robot based on previous performance;

Sense the environment and get sensor inputs S_1_,S_2_…S_10_;

Creating signaling agents based on the sensor inputs;

Activated all extant agents in the robot;

Referee the interaction of all agents;

Update the genes and agents;

Check the output signals: T_1,_T_2…._T_14_;

Use the output signals to set the parameters of actuators;

Check whether the robot has enough energy to perform the behavior of actuators;

If (energy is ok)

Then implement the behavior and cost energy;

Else

Do nothing;

Check E and record behavior of the robot;

Calculate the fitness of the robot for next step;

If(E>0)

Record the information of every robot and output them as the result at that time step;

Else

Call Delete function;

}

# Appendix B: Additional details about the agent’s function in the GRN controller.

- The Init() function reads the interaction rules from the corresponding gene and load this knowledge into the memory of the new agent.
- The decay() function decreases the concentration of the agent based on the situation at the last time step.
- The Sense() function updates the current situation, which include the change on performance feedback, total number of agents in the GRN, concentration level and energy level.
- The ReadGenome() function checks the whole genome for finding ‘matching’ genes and then invokes a Bind() function when the gene is found. This function will decrease the concentration of the agent and stops when Binding has occurred. This function can iterate multiple times based on the concentration level of the agent.
- The Bind() function checks whether the gene/agent could bind with another gene/agent or actuator; if it can, the agent will ‘regulate’ the behavior of that gene/agent or output a signal to the actuator
- The Interact() function will check whether the current agent affects the concentration of other agents. Such interaction is based on the binding motif of the agent.
- The Feedback() function send feedback to the gene in the genome (influences gene expression), based on the agent’s average fitness
- The Remove() function will delete the agent and release the memory.
